# Two causes of palpitations, detected by photoplethysmography on a mobile phone

**DOI:** 10.1007/s12471-018-1208-z

**Published:** 2018-11-27

**Authors:** W. Gielen, M. Gielen

**Affiliations:** Silkeborg Regional Hospital, Silkeborg, Denmark

A 54-year-old woman, treated for hypertension, visits her general practitioner with symptoms of recurrent palpitations. Earlier Holter monitoring has been normal except for premature atrial contractions. Her symptoms occur spontaneously and are not stress-related. Physical examination and ECG were without abnormalities. To document her symptoms over a longer period of time, her general practitioner prescribed the FibriCheck app for the duration of a month, which she could download onto her mobile phone. In case of any symptoms, she had to put her finger on her phone’s camera. This allowed FibriCheck to record the rhythm for a duration of 60 s using photoplethysmography (PPG), a simple optical technique used to detect volumetric changes in the blood in the peripheral circulation (Figs. 1 and 2; [1–3]). The results were automatically analysed by the algorithm, then sent to, analysed and validated by a local cardiologist. With symptoms three kinds of rhythms were observed.

**Fig. 1:**
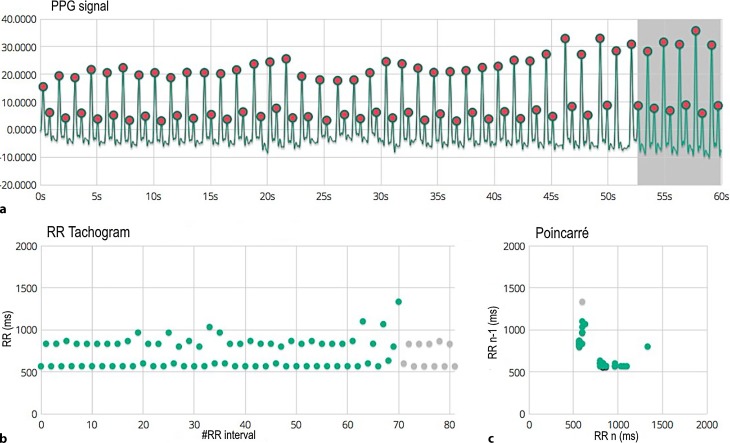
Photoplethysmogram (PPG) recording. **a** 60 s PPG signal measured at the patient’s fingertip with the phone camera. **b** RR tachogram which represents the distance between the RR intervals in milliseconds. **c** The Poincaré plot shows how the present RR interval is related to the previous one

**Fig. 2:**
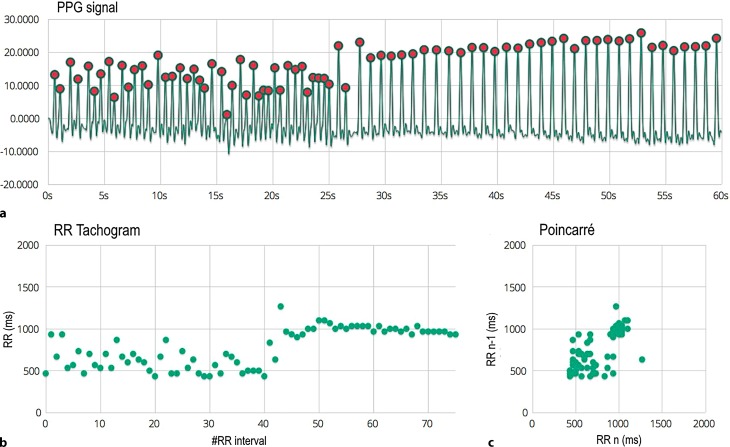
Photoplethysmogram (PPG) recording. **a** 60 s PPG signal measured at the patient’s fingertip with the phone camera. **b** RR tachogram which represents the distance between the RR intervals in milliseconds. **c** The Poincaré plot shows how the present RR interval is related to the previous one

Which rhythms can be recognised on these PPG recordings?

## Answer

You will find the answer elsewhere in this issue.
